# New Visualization Models of Designation Pathway and Group Categorization of Device–Drug and Device–Biologic Combination Products Classification in the United States: Analysis of FDA Capsular Decisions

**DOI:** 10.1007/s43441-021-00276-x

**Published:** 2021-04-12

**Authors:** Nobuo Uemura, Hiroshi Kasanuki, Mitsuo Umezu

**Affiliations:** 1 Joint Graduate School of Tokyo Women’s Medical University and Waseda University (TWIns), Cooperative Major in Advanced Biomedical Sciences, Graduate School of Advanced Science and Engineering, Waseda University, Tokyo, Japan; 2 Waseda Institute for Medical Regulatory Science, Waseda University, Tokyo, Japan; 3 Department of Integrative Bioscience and Biomedical Engineering, Graduate School of Waseda University, Tokyo, Japan; 4 Joint Graduate School of Tokyo Women’s Medical University and Waseda University (TWIns), Cooperative Major in Advanced Biomedical Sciences, Graduate School of Advanced Science and Engineering, Waseda University, c/o UMEZU Lab, 2-2 Wakamatsucho, Shinjukuku, Tokyo, 162-480 Japan

**Keywords:** Classification, Combination product, Medical device, Drug/biologic, Regulations

## Abstract

**Objective:**

The developer and sponsor of new combination products in US needs to forecast which classification and designation to the regulatory scheme of drug, biological product, or device would be required for the new products by the Food and Drug Administration (FDA).

To improve the predictability and acceptability of the designation of new combination products for innovators, developers, and sponsors, and to encourage the development and early access of new combination products, we proposed new visualization models of the designation pathway and group categorization.

**Method:**

We searched the website of the FDA on 15 November, 2020 to identify the regulatory scheme of the FDA’s 129 capsular decision cases of device–drug and device–biologics combination products and other publicly available cases the FDA designated to the drug/biologic or device regulatory scheme.

**Results:**

By introducing a new definition for primary intended use (PIU) by developers and sponsors extracted from the classification factors of primary mode of action (PMOA), we developed new visualization models of the designation pathway and two-dimensional group categorization. And applying these models to the cases the FDA designated, we proposed a new group categorization of combination products while focusing on the device component function.

**Conclusions:**

The new visualization models with PIU and PMOA and the new group categorization focusing on the device component function proposed in this study may increase predictability and acceptability of the classification of newly developed combination products into the regulatory scheme of drug, biological product, and device, for innovators, developers, and sponsors.

## Introduction

The United States Food and Drug Administration (FDA) regulates medical products in US and defines combination products in 21 Code for Federal Regulations 3.2(e) and the term ‘combination product’ includes (1) a product (single entity) comprised two or more regulated components; (2) two or more products packaged together (co-packaged); (3) separately packaged products; or (4) an investigational product intended for use only with a specified product (cross-labelled), such as drug/device, biologic/device, drug/biologic, or drug/device/biologic [1–3].

According to the product’s primary mode of action (PMOA), the FDA assigns combination product submissions to one of the following centers, which acts as the lead center: Center for Drug Evaluation and Research (CDER), Center for Biologics Evaluation and Research (CBER), or Center for Devices and Radiological Health (CDRH) [3–5].

**Figure 1:**
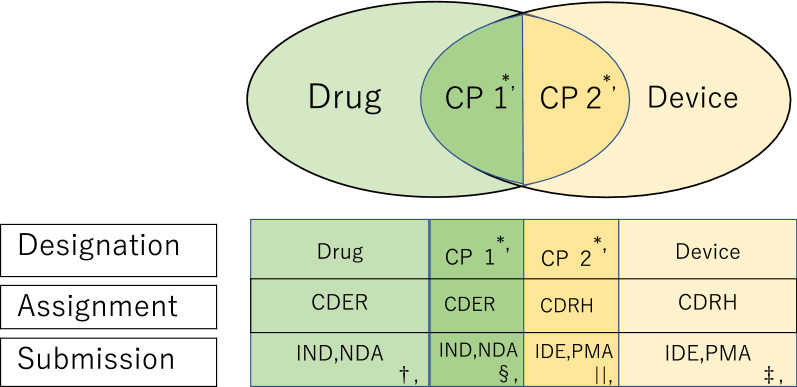
US FDA’s Classification and Assignment of Drug, Device, and Drug–Device Combination Products (CP). *, CP 1 is a combination product under drug regulatory scheme, whereas CP 2 is under device regulatory scheme. †, Submission (IND, NDA) of the new product designated as drug by FDA is assigned to CDER. Submission (IND, BLA) of the new product designated as biological product by FDA is assigned to CBER (some biological products, such as proteins, are assigned to CDER). ‡, Submission (IDE, PMA) of the new product designated as device by FDA is assigned to CDRH. §, Submission (IND, NDA) of the new product designated as combination product by FDA is assigned to CDER when the drug constituent part provides the primary mode of action. Submission (IND, BLA) of the new product designated as combination product by FDA is assigned to CBER (some product to CDER) when the biological product constituent part provides the primary mode of action. ||, Submission (IDE, PMA) of the new product designated as combination product by FDA is assigned to CDRH when device constituent part provides the primary mode of action

The new drug–device or biologic–device combination product designated to the drug or biologic product regulatory scheme and assigned to CDER or CBER should follow the procedures for investigational new drug (IND) and new drug application (NDA) or biologics license application (BLA), respectively, whereas those designated to the device scheme and assigned to CDRH should follow the procedures for investigational device exemption (IDE) and premarket approval application (PMA) procedure (Fig. 1).

Therefore, the developer and sponsor of new combination products in US need to forecast, at the early stage of product development in advance to the consultation and submission process with the FDA, which classification and designation to the regulatory scheme of drug, biological product, or device would be required for the new products by the FDA at the submission of application. Predictability of classification is important for developers and sponsors to prepare the pre-clinical and clinical evaluation data in the application dossier, and the quality and safety management of new combination products [2].

FDA’s guidance documents provide information on combination products for developers and sponsors at the development, submission, and dispute process, regarding the Classification, Request for Designation, Pre-Request for Designation, Early Development Consideration, and Submission and Resolution of Disputes [6–9]. The Request for Designation (RFD) and the Pre-Request for Designation (pre-RFD) by developer and sponsor to the FDA were introduced to obtain the determination or informal feedback from the FDA, and a new guidance document titled “Requesting FDA Feedback on Combination Products” explains the sponsor best practices and available feedback mechanism [10].

The FDA decides the classification and designation of individual cases of combination product on a case-by-case basis based on the PMOA, disseminating the list of capsular decisions regarding the combination product assignments on the FDA website [11, 12].

Additionally, the FDA presents the performance report as ‘the combination products performance report to the Congress for the Office of Combination Products (OCP)’, which covers the activities and accomplishments related to the application submission, consultation request, center assignment, premarket review, and post-market regulation, including workload by application type [13].

The FDA emphasizes that the PMOA of a combination product is the key factor for its designation and assignment. However, sometimes FDA’s case-by-case decision on the designation and assignment of a new combination product may not be predictable or acceptable for developer and sponsor, sometimes the designation of new combination products applications had changed from device to drug regulatory scheme, and in some cases, the regulatory decisions by the FDA regarding the classification and jurisdiction assignments as drug may be uncertain or in dispute in the case of the pressurized canister with a chemical neutralization drug [5–7, 12].

We postulated that visualizing the decision pathway and the position of the group of drug–device or biologic–device combination products may help innovators, developers, and sponsors to forecast the classification and designation of newly developed combination products into either drug/biologic or device regulatory schemes, even at an early stage of the product development, and before preparing the pre-clinical and clinical evaluation data in the application dossier and consulting with the FDA.

In this article, we introduced a new definition for primary intended use (PIU) of the combination product by developers and sponsors which was extracted from the classification factors of primary mode of action (PMOA), and proposed new models for the designation pathway and the group positioning of the combination products to visualize the group of categories and the classification falling into either drug/biologic or device regulatory schemes, that could increase the predictability and acceptability of the classification of newly developed combination product for the innovators, developers, and the sponsors.

## Methods

### Analysis of FDA/OCP Performance Report

Using the FDA website on the FDA/OCP Performance Report for the fiscal year (FY) 2011 to FY 2019, we analyzed the publicly available data about the number of the combination products which the FDA has designated and classified, by the original NDAs, BLAs, PMAs, and Humanitarian Device Exemptions (HDEs), and by their group types [13].

### New Definition of PIU

Utilizing the information requested from the sponsors by the FDA in the Request for Designation (RFD) [6] or Pre-RFD [7] to designate the new combination product, we extracted a factor of the intended use (IU) and introduced the PIU of the combination product which will be distributed in the market. PIU is one of the factors we have proposed in the new model.

### Proposal for a New Model of Designation Pathway

Using the PIU and PMOA, we proposed a new model of the designation pathway for combination products to visualize the two-step flow of designation of a product falling into either the drug/biological or device regulatory scheme.

We introduced the scores (*r*) and (*e*) for the drug/biologic and device components of combination products and the *I*-score and *M*-score for PIU and PMOA, respectively, to identify the flow of case direction in the model. In the case of *I*-score of PIU, Drug/Biologic component (*r*) had one of the scores of r2: Principal, r1: Ancillary, r0: No therapeutic meaning, as well as Device component (*e*) of e2: Principal, e1: Ancillary, e0: No therapeutic meaning, and *I*-score consisted of the combination of scores of (*r*) and (*e*); for example, r2e1: Principal for drug and ancillary for device.

In the case of *M*-score of PMOA, Drug/Biologic component (*r*) had one of the scores of r2: Principal, r1: Ancillary, r0: No therapeutic contribution, as well as Device component (e) of e2: Principal, e1: Ancillary, e0: No therapeutic contribution, and *M*-score consisted of the combination of scores of (*r*) and (*e*); for example, r2e0: Principal for biologic and no therapeutic contribution for device. We analyzed 129 cases of the FDA’s Capsular Decision of Drug–Device and Biologic–Device Combination Product Assignments, publicly available on the FDA website [11]. We did not analyze nine cases of drug–biologic combination products because of the lack of device components in the products. Furthermore, we analyzed other classification difficult cases publicly available on the website.

### Proposal of the New Two-Dimensional Model of Group Positioning and New Categorization, and Analysis of FDA’s Capsular Decision of Assignments

By separating the factors for the IU and mode of action (MOA) and using the *I*- and *M*-scores, we proposed the new two-dimensional model for group positioning of device–drug and device–biologic combination products. To obtain the group positioning in the new model and group of categories focusing on the device component function, we analyzed 129 cases of the FDA’s capsular decision and other classification difficult cases, publicly available on the FDA website.

### No Patient and Public Involvement

This research was performed without patient involvement. Patients were neither invited to comment on nor contribute to the study. However, we consider that these results could improve patients and public understanding of the classification of combination products.

## Results

### Analysis of FDA/OCP Performance Report

**Figure 2:**
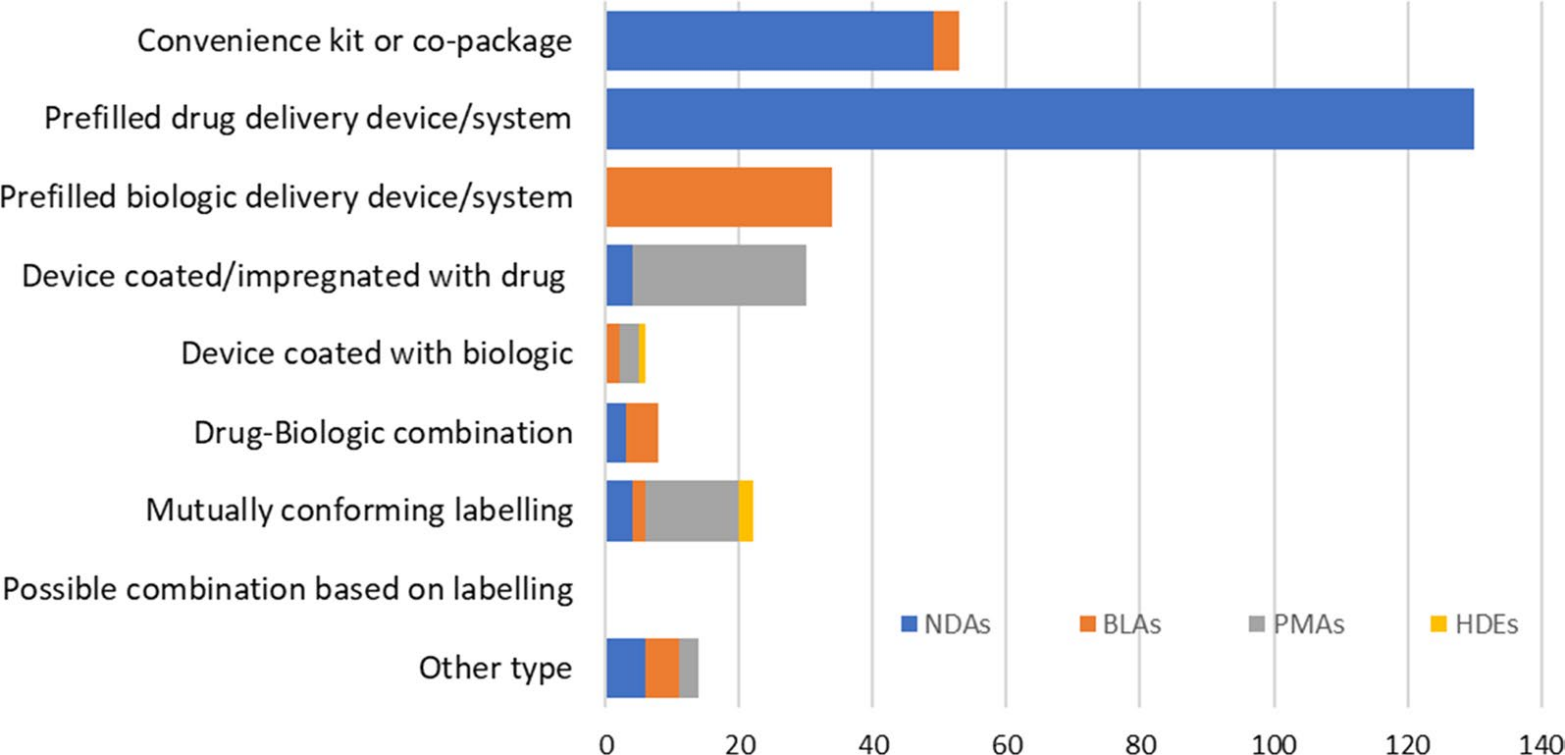
Combination Products classified (FY2011–FY2019) Number of Original NDAs, BLAs, PMAs and HDEs. *, Type and its Description (1) Convenience Kit or Co-Package Drug and device are provided as individual constituent parts within the same package. (2) Prefilled Drug Delivery Device/System Drug is filled into or otherwise combined with the device and the sole purpose of the device is to deliver drug. (3) Prefilled Biologic Delivery Device/System Biological product is filled into or otherwise combined with the device and the sole purpose of the device is to deliver biological product. (4) Device Coated/Impregnated/Otherwise Combined with Drug Device has an additional function in addition to delivering the drug. (5) Device Coated or Otherwise Combined with Biologic Device has an additional function in addition to delivering the drug. (6) Drug/Biologic Combination. (7) Separate Products Requiring Cross Labelling. (8) Possible Combination Based on Cross Labelling of Separate Products. (9) Other Type of Part 3 Combination Product (e.g., Drug/Device/Biological Product) Combination product not otherwise described

FDA/OCP performance reports provide the number of combination products that the FDA classified in each FY via original NDAs, BLAs, PMAs, and HDEs [13]. These include the drug–device, biologic–device, and drug–biologic combination products. The accumulation of the number of classified products in the reports from FY 2011 to FY 2019 was shown in Fig. 2. The classified combination products in each type (from 1 to 9) were divided based on the submission types of the designated classifications, which were based on the product’s PMOA.

The types 4, 5, 7, and 9 included the cases that fell into both the drug/biologic and device regulatory schemes. And in the case of types 2, 3 for example, some applications initially designated as device regulatory scheme in the performance report had changed to drug/biologic regulatory scheme as the confirmed application in the next year performance report.

### New Definition of PIU

To consider the PMOA for classification and designation, the FDA requests the following basic information in the RFD or Pre-RFD of the combination products:

- Description of the product.
- A listing of all the components/ingredients.
- An explanation of how the product works.
- All known MOAs and the mechanism(s).
- Instructions for use/conditions of use.
- Proposed use/intended use/indications for use statement [6, 7, 14].

The definitions of MOA and PMOA of a combination product, described in the Code of Federal Regulation [5] are as follows:

A mode of action (MOA) is defined as the means by which a product achieves its intended therapeutic effect or action. The product may be a drug, biological product, or device mode of action.

Primary MOA (PMOA) is defined as the single mode of action of a combination product that provides the most important therapeutic action of the combination product.

Then, we extracted information regarding the intended use (IU) by developers and sponsors, and introduced the new definition of the PIU of the final combination product as the most important single IU of the product developed and distributed in the market. The PIU of the newly developed product depends on the developer’s and sponsor’s intention and judgement of the proposed IU of either the drug/biologic or device, while the PMOA depends on scientific evaluation by the developer/sponsor and regulatory authority.

### Proposal for the New Model of Designation Pathway (Combination Product Designation Pathway Model: CPDP Model)

We recognized that in considering the regulatory scheme categorization of the new combination product, the PIU of the final product was an important factor for its designation to the drug/biologic or device regulatory scheme, prior to considering the PMOA.

**Figure 3:**
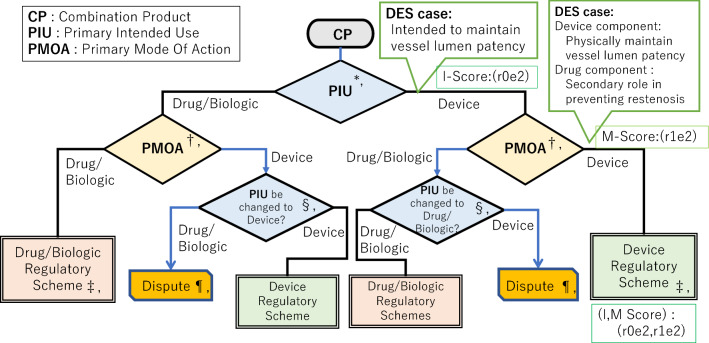
New Model of Designation Pathway for Device–Drug and Device–Biologic Combination Products’ Categorization (Case application of Drug Eluting Stent (DES) to the CPDP model). *, At the first step the Primary Intended Use (PIU) of Combination product (CP) as final whole product is assessed whether the direction of *I*-score of PIU is either drug/biologic or device. *I*-score consists of the drug/biologic component (*r*) and the device component (*e*) as principal (2), ancillary (1), or no meaning (0) for IU. †, At the second step the Primary Mode of Action (PMOA) of CP is assessed whether the direction of is *M*-score of PMOA is either drug/biologic or device. *M*-score consists of the drug/biologic component (*r*) and the device component (*e*) as principal (2), ancillary (1), or no contribution (0) for MOA. ‡, When the directions of first and second assessment are coincident as either drug/biologic or device, designation falls into that classification. §, When the first and second assessment are inconsistent, alternate Intended Use should be considered whether the *I*-score of sponsor’s PIU can be change. ||, Blue arrow is a case when sponsor’s initial PIU and sponsor’s and regulatory authority’s recognition of PMOA are different. ¶, When the sponsor and regulatory authority have not reached the common coincident classification, dispute will occur and the development of the new combination product will be discontinued. **, Green box is a case application of Drug Eluting Stent (DES) to the model with its *I*-, *M*- and *I*, *M*-scores. ††, *I*-score of Intended Use for each component is either Principal (2), Ancillary (1) or No therapeutic meaning (0). *I*-score of PIU falls into the following 4 patterns; r2e0: Drug Component (*r*): Principal (2), Device Component (*e*): No meaning (0) ⇒ Left, r2e1: Drug Component (*r*): Principal (2), Device Component (*e*): Ancillary (1) ⇒ Left, r1e2: Drug Component (*r*): Ancillary (1), Device Component (*e*): Principal (2) ⇒ Right, r0e2: Drug Component (*r*): No meaning (0), Device Component (*e*): Principal (2) ⇒ Right. ‡‡, *M*-score of Mode of Action for each component is either Principal (2), Ancillary (1) or No therapeutic contribution (0). *M*-score of PMOA falls into the following 5 patterns; r2e0: Drug Component (*r*): Principal (2), Device Component (*e*): No meaning (0) ⇒ Left, r2e1: Drug Component (*r*): Principal (2), Device Component (*e*): Ancillary (1) ⇒ Left, r2e2: Drug Component (*r*): Principal (2), Device Component (*e*): Principal (2) ⇒ Same as *I*, r1e2: Drug Component (*r*): Ancillary (1), Device Component (*e*): Principal (2) ⇒ Right, r0e2: Drug Component (*r*): No meaning (0), Device Component (*e*): Principal (2) ⇒ Right. ***, (*I*, *M*) Score of PIU and PMOA varies following 8 patterns; (r2e0, r2e0), (r2e0, r2e1), (r2e1, r2e1), (r2e1, r2e2), (r1e2, r2e2), (r1e2, r1e2), (r0e2, r1e2), (r0e2, r0e2)

We proposed a simple, symmetric, and new visualization model of designation pathway for device–drug and device–biologic combination products into the drug/biologic and device regulatory scheme (Fig. 3); the pathway comprised a two-step procedure involving (1) PIU and (2) PMOA.

To easily apply the PIU and PMOA to the drug/biologic (left direction) or device (right direction) side in the flow in Fig. 3, we introduced the *I*-score and the *M*-score using scores of the drug/biologic component (*r*) and the device component (*e*) as the principal (2), ancillary (1), or no meaning (0) for IU, and as principal (2), ancillary (1), or no contribution (0) for MOA.

The *I*-score of the PIU varied as (r2e0), (r2e1), (r1e2), and (r0e2) as one of the components was principal (2) and the other was considered no meaning (0) or ancillary (1).

The *M*-score of the PMOA varied as (r2e0), (r2e1), (r2e2), (r1e2), and (r0e2) because one of the components was principal (2) and the other was either no contribution (0), ancillary (1), or principal (2), which had dual MOA.

The two-step procedure introduced in the new model of designation pathway was followed by the left direction in (r2e0) or (r2e1), and the right direction in (r1e2) or (r0e2) to fall into the regulatory scheme. When the *M*-score was (r2e2), the second step followed the same direction as the first step of the *I*-score.

When the two-step directions were coincidental, the designation leads to regulatory schemes such as drug/biologic or device. When these were inconsistent, alternate PIU should be re-considered by the developer and the sponsor to reach the coinciding designation.

To verify the achievability of the new model which we named Combination Product Designation Pathway model (CPDP model) as shown in Fig. 3, we examined 129 cases of capsular decision available on the FDA website.

In the case of (1), prefilled syringe, *I*-score and *M*-score were (r2e0), leading to a drug regulatory scheme. In the case of (2) and (3), a stent eluting drug and bone void filler with an antibiotic, respectively, the *I*-score was (r0e2) and *M*-score was (r1e2), resulting in device regulatory scheme. The case application for the drug-eluting stent was shown in Fig. 3.

Furthermore, we examined certain cases that were difficult to classify and were available on the website. In the case of 94), which included contact lenses with a drug for the treatment of glaucoma, the MOAs of both the drug and device components were dual, and the *M*-score was (r2e2). Therefore, the designation depended on the direction of the *I*-score, resulting in the drug regulatory scheme. In the case of (5), which included a pressurized canister with a chemical neutralization drug, the initial PIU by the sponsor led to the device inception; however, the FDA judged the PMOA as a drug. These inconsistencies were disputed, and the case of the pressurized canister with a chemical neutralization drug which was designated as drug in US despite labelled as device in EU was resulted in a court trial [15].

### Proposal of the New Two-Dimensional Model of Group Positioning and New Categorization (Combination Product 2-Dimensional Model: CP2D Model), and Analysis of the FDA’s Capsular Decision of Assignments

**Figure 4:**
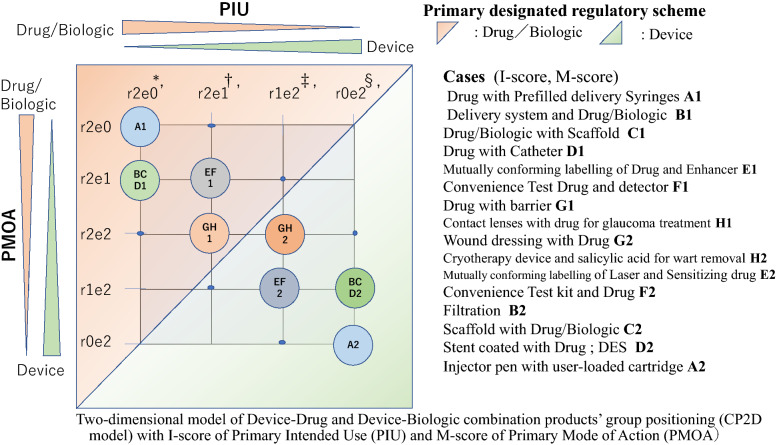
Two-dimensional model of Device–Drug and Device–Biologic combination products’ group positioning (CP2D model) with *I*-score of Primary Intended Use (PIU) and *M*-score of Primary Mode of Action (PMOA). *, When the *I*-score of PIU is (r2e0), *M*-score is either (r2e0) or (r2e1) because (r2e2) of *M*-score must lead ancillary IU of one of the components which cannot be no meaning of IU. †, When the *I*-score of PIU is (r2e1), *M*-score is either (r2e1) or (r2e0) because (r2e0) means no contribution of device component and it cannot bring ancillary IU of device component. ‡, When the *I*-score of PIU is (r1e2), *M*-score is either (r1e2) or (r2e2) because (r0e2) means no contribution of drug/biologic component and it cannot bring ancillary IU of drug/biologic component. §, When the *I*-score of PIU is (r0e2), *M*-score is either (r1e2) or (r0e2) because (r2e2) of *M*-score must lead ancillary IU of one of the components which cannot be no meaning of IU

By separating the factors of PIU and PMOA during the designation pathway in the model, we proposed the new two-dimensional visualization model of group positioning for device–drug and device–biologic combination products, which we named Combination product 2-Dimensional model (CP2D model) as shown in Fig. 4.

The model presented vertical and horizontal axes of PIU and PMOA with 4 grades of *I*-score and 5 grades of *M*-score.

In Fig. 3, the model of the designation pathway (*I*, *M*) scores varied into eight patterns: four groups, (r2e0, r2e0), (r2e0, r2e1), (r2e1, r2e1), and (r2e1, r2e2) were designated to the drug/biologic regulatory scheme, whereas the other four groups, (r1e2, r2e2), (r1e2, r1e2), (r0e2, r1e2), and (r0e2, r0e2) were designated to the device scheme.

We analyzed the above eight groups of the (*I*, *M*) score and the positions in the model in which the FDA’s 129 cases of Capsular Decision and other classification of difficult cases, mentioned in the previous section, were distributed.

While considering the form and the function of the device component in the case of combination products, we also established the eight groups of categories focusing on the device function from A to H, as shown in Table 1.

**Table 1:** New Categorization of Device–Drug and Device–Biologic Combination Products Based on the Two-Dimensional Model of Combination Product Group Positioning with *I*-Score of Primary Intended Use (PIU) and *M*-Score of Primary Mode of Action (PMOA)

PIU	PMOA	Group of Devices*	Device Function	Example
r2e0	r2e0	A Injector^†^	A1 Delivery	A1 Drug with Prefilled delivery Syringes
r2e0	r2e1	B Delivery + Filtration etc.^‡^	B1 Delivery +	B1 Delivery system and Drug/Biologic
r2e0	r2e1	C Scaffold, Implant, etc.^‡^	C1 Delivery +	C1 Drug/Biologic with Scaffold
r2e0	r2e1	D Catheter, Stent, etc.^‡^	D1 Delivery +	D1 Drug with Catheter
r2e1	r2e1	E Light, Laser, etc.^§^	E1 Enhance	E1 Drug and Enhancer
r2e1	r2e1	F Test Device^||^	F1 Detect	F1 Convenience Test Drug and detector
r2e1	r2e2	G Dressing, Barrier, etc.^¶^	G1 Barrier	G1 Drug with barrier
r2e1	r2e2	H Independent**	H1 Dual	H1 Contact lenses with drug for glaucoma treatment
r1e2	r2e2	H Independent**	H2 Dual	H2 Cryotherapy device and salicylic acid for wart removal
r1e2	r2e2	G Dressing, Barrier, etc.^¶^	G2 Barrier	G2 Wound dressing with Drug
r1e2	r1e2	F Test Device^||^	F2 Test	F2 Convenience Test kit and Drug
r1e2	r1e2	E Light, Laser, etc.^§^	E2 Enhanced	E2 Laser and Sensitizing drug
r0e2	r1e2	D Catheter, Stent, etc.^‡^	D2 Assisted	D2 Stent coated with Drug; DES
r0e2	r1e2	C Scaffold, Implant, etc.^‡^	C2 Assisted	C2 Scaffold with Drug/Biologic
r0e2	r1e2	B Delivery + Filtration etc.^‡^	B2 Filtration	B2 Filtration
r0e2	r0e2	A Injector^†^	A2 Delivery	A2 Injector pen with user-loaded prefilled cartridge

Note (1) Regulatory Scheme applied either (X1): Drug/Biologic or (X2): Device

Note (2) Example of H1 and A2 are classification difficult cases

*Group of devices

Group A ‘Injector’ includes Syringes, Cartridges, Injector, Applicator

Group B ‘Delivery system + Filtration etc.’ includes Delivery device, Delivery system, Inhaler, Separation, Filtration system, Dialysate, Flush solution

Group C ‘Scaffold, Implant, etc.’ includes Scaffold, Bone void filler, Matrix, Dental implant

Group D ‘Catheter, Stent, etc.’ includes Cardiovascular stent, Coronary stent, Vascular graft, Catheter, Respiratory, Tube, Varnish, Dental material, Dental device, Dental floss, Wax, Paste, Sealant, Cleanser

Group E ‘Light, Laser, etc.’ includes Light, Laser, Fluorescent, Chemi-luminescent, Ultrasound, Radiation, Imaging system

Group F ‘Test Device’ includes Test kit, Detector, Monitor, and Production, collection, or analysis device

Group G ‘Dressing, Barrier, etc.’ includes Bandage, Wound dressing, Embolization device, Granule, Mesh, Swab, Barrier, Tampon, Condom

Group H Independent (Dual function) includes Contact lenses, Cryotherapy device

^†^Group (A): combination product with Injector device component has score r2e0, r2e0 when injector component has a drug-delivery function, but it has score r0e2, r0e2 when injector component can be loaded by several kinds of drug cartridges separately

^‡^Group (B): combination product with delivery system has score r2e0, r2e1 when delivery system component has a drug-delivery and other additional function such as filtration, but it has score r0e2, r1e2 when filtration system can be applied to several kinds of drug/biologics

Group (C): combination product with scaffold etc. has score r0e2, r1e2 when scaffold component is assisted by the drug/biologics component, but it has score r2e0, r2e1 when scaffold is used as drug/biologics delivery device

Group (D): combination product with catheter etc. has score r0e2, r1e2 when catheter component is assisted by the drug component for prevention of thrombosis, but it has score r2e0, r2e1 when catheter is used as drug-delivery device

^§^Group (E): combination product with laser etc. has score r2e1, r2e1 when laser component enhance the drug activity, but it has score r1e2, r1e2 when laser function is enhanced by the drug component

^||^Group (F): combination product with test device has score r2e1, r2e1 when test device component detect the drug activity, but it has score r1e2, r1e2 when device test function is assisted by the drug component

^¶^Group (G): combination product with dressing etc. has score r2e1, r2e2 when dressing component has a function as barrier to enhance the drug activity, but it has score r1e2, r2e2 when dressing healing function is enhanced by the drug component

**, ^††^Group (H): combination product with dual function of drug/biologics and device has score r2e1, r2e2 when the intended use (example: treatment of glaucoma) is as drug, but it has score r1e2, r2e2 when intended use (example: cryotherapy by device) is as device

The device function of groups A1 to D1 was ancillary delivery of the drug/biologics, while that of the groups A2 to D2 was principal delivery, filtration, and the device’s own functions assisted by the drug components.

The device function of group E2 was enhanced by the drug component.

Groups F2, G2, and H2 were classified into the device regulatory scheme because their device component had principal functions.

Each group may include (1) single-entity, (2) co-packaged, and (3) cross-labelled type of combination products.

The visualization model in Fig. 4 and the new group categories presented in Table 1 depict the case examples with their (*I*, *M*) scores derived from the PIU and PMOA. Even in case of the same group categories, the combination products were designated to either drug/biologic or device regulatory schemes depending on the PIU, PMOA, and (*I*, *M*) scores in the model.

## Discussion

The FDA has regulatory designation and definitions for the ‘combination product’, as well as for ‘drug’, ‘biological product’, and ‘medical device’ [1]. However, the FDA needs to allocate and assign the new combination product submission to one of the regulatory schemes of ‘drug’, ‘biological product’, and ‘medical device’, and assign the application to one of the leading centers for reviewing: CDER, CBER, or CDRH.

The FDA has elaborated that designation and categorization are based on the PMOA of the combination product submitted or to be submitted to the FDA [16]. The FDA accumulates case-by-case decisions of designation and provides the list of capsular decisions on designation and assignment of new combination products to the leading centers for reviewing [11]. Furthermore, the FDA also provides jurisdictional updates and related information on its website [17].

Recently, the FDA has issued a proposed rule regarding product jurisdiction to clarify the scope of regulation and procedures for requesting product classification as drugs, biological products, devices or combination products [18], and guidance document for the best practices regarding the interactions between the FDA and the sponsors for combination products [10].

However, in the case of newly developed combination products, the classification and designation are not necessarily certain to the innovators, developers and the sponsors towards market authorization.

The FDA mainly considers the reviewer’s point of view on the safety, quality, and efficacy of the new product at the premarket evaluation of the application. However, the market authorization holders and product users believe that the characterization and IU of the new combination products as medical diagnostics or patient treatments are important in the market.

In the case of a device–drug or a device–biologic combination product, incorporation of the new definition of PIU, derived from IU extracted from the designation information factors, resulted in the categorization by the new visualization model of the designation pathway and group positioning to either drug/biologic or device regulatory schemes. Unlike PMOA, the PIU of the product depends on the intention of the developer and sponsor about the product potential and the value for medical diagnosis and treatment, as well as on the perspective of not only the innovators, developers and sponsors but also the users, such as physicians and patients.

Occasionally, sponsors prefer the regulatory scheme with cheaper submission fees and probably faster reviewing times for new products. However, the intensions of these sponsors may sometimes cause disputes because of varying opinions on classification as opposed to the FDA’s scientific decision from the regulatory authorities’ point of view, mostly regarding mismatch between the PIU by the developer and sponsor and the PMOA judged by the authority.

The FDA has a process to settle any dispute between the sponsor and the authority regarding the classification of newly submitted products [9]. However, the new visualization models with PIU and PMOA may increase the predictability and acceptability of the classification for the innovators, developers, and sponsors at an early stage of the product development in advance to the process of consultation with and submission to the FDA. A classification consensus among developers, sponsors and regulators may encourage the development and early access of new combination products for efficiently preparing the pre-clinical and clinical evaluation data, and may boost the value and benefit of the new combination products for patients by increasing their sense of confidence regarding the safety and the effectiveness throughout the life cycle of the product. We desired that this article could inspire the application of the models by the professionals of developers and sponsors, and their dialogue with authorities.

### Limitations of this Study

New visualization models proposed in this study can be applied to the cases provided by the FDA as capsular decisions, and we analyzed only the cases which were publicly available on the website. We could not analyze the cases of combination products under development, investigation, or reviewing by the FDA.

In this study, we did not analyze cases of combination products or combined products with drug and device components in other countries and jurisdictions. The regulatory schemes of combination products in the US differ from the schemes in Japan, UK, and the member states of the EU. Moreover, the new European Union Medical Device Regulation is changing the regulatory scheme on device-medicinal product combinations [19–22].

The regulatory schemes in the US, EU, and Japan are shown in Table 2.

**Table 2:** Regulation of Combination Product (CP): Application and Reviewing—Once Classified as ‘medical device application’, Device Regulatory Scheme Applies to the Product

	USA	EU	Japan
Definition: component and CP	Drug	Medicinal Product (MP), Advanced Therapy Medicinal Product (ATMP)	Drug (pharmaceuticals),
	Biologic		Regenerative Medical Product
	Device	Medical device (MD)	Medical Device
	CP	CP as medicinal product, CP as medical device, Combined ATMP	CP as drug, CP as medical device
Scope/range of CP	Single entity	Integral	Single product applied for approval
	Co-package	Co-packaged	NA
	Cross-labelled	Separately obtained and referred to	NA
Agency/review determination	FDA: Leaded by CDER/CBER/CDRH	MP: EMA/CNA	MHLW/PMDA/Assigned office
		MD: NB (conformity assessment)	
Application (Appl.)	NDA, BLA	Medicinal product Appl	Pharmaceuticals Appl
	PMA, HDE, 510 k	Medical Device Appl	Medical Device Appl
User fee (application fee, as of April 2019)	Drug NDA: $ 2,588,478	Medicinal Product (MP) to EMA: 291,800 EURO	New Drug (New API to PMDA): ¥46,901,700
	Biologic BLA: $ 322,147		Regenerative Medical Product (new) to PMDA: ¥20,279,600
	Device PMA: $ 322,147	Medical device (MD) to NB: example Ca 6500 EURO	New Medical Device (to PMDA): ¥17,721,200 for Class IV, ¥13,016,900 for Class III

*CP* combination product, *Appl* Application, *NDA* new drug application, *BLA* biologics license application, *PMA* premarket approval, *HDE* humanitarian device exemption, *MP* medicinal products, *MD* medical device, *ATMP* advanced therapy medicinal product, *cATMP* combined advanced therapy medicinal product, *EMA* European Medicine Agency, *CAN* competent national authority, *NB* notified body, *API* active pharmaceutical ingredient, *PMDA* Pharmaceutical and Medical Device Agency

However, even with the differences in the definition of combination products and the regulatory schemes among the US, EU, and Japan, the concept of PIU and PMOA can be convergent, and the new models shown in this study could be applied to cases in the EU and Japan. Further studies about the cases in other countries and jurisdictions may contribute to expand the feasibility and usefulness of new models for increasing the predictability of classification of new combination products during the development in other countries.

Furthermore, regulatory authorities in some other countries have a classification for borderline products, which include not only combination, but also single products, which are difficult to be designated to drug, biologics or device. But these models may be applicable to both the combination products and single-entity borderline products.

To facilitate international convergence, extensive information on PIU and PMOA and sharing experiences regarding the designation by regulatory authorities in different countries and jurisdictions must be necessary and important.

The new visualization model of the designation pathway for the categorization of combination products reported in this article may be applicable to innovative combination products of new conceptual and mechanistic constituent parts with novel technologies, such as drug combined with device and the Software as Medical Device (SaMD), Advanced Therapeutic Products (ATP) including device components, and medical devices composed of new materials. In these cases, however, further understanding the description, safety, and efficacy of the new products and investigating their PIU and PMOA are necessary to forecast the classification and the regulatory schemes for these products.

## Data Availability

All data relevant to this study are publicly available on the FDA websites. Capsular Descriptions of Jurisdictional Determinations (https://www.fda.gov/CombinationProducts/JurisdictionalInformation/RFDJurisdictionalDecisions/default.htm).
